# Structural and physicochemical stability of 3D-printed bolus materials used in radiotherapy

**DOI:** 10.1038/s41598-026-36952-x

**Published:** 2026-01-29

**Authors:** Karolina Jezierska, Martin Borůvka, Martina Ryvolová, Adam Hotař, Helena Gronwald, Magdalena Łukowiak, Piotr Rawojć, Helena Rudnicka, Totka Bakalova

**Affiliations:** 1 Department of Medical Physics, Pomeranian Medical University in Szczecin, ul. Ku Słońcu 13, 71-073 Szczecin, Poland; 2 Department of Engineering Technology, Faculty of Mechanical Engineering, Technical University of Liberec, Studentska 2, 461 17 Liberec 1, Czech Republic; 3 Department of Material Science, Faculty of Mechanical Engineering, Technical University of Liberec, Studentska 2, 461 17 Liberec, Czech Republic; 4 Department of Propedeutics, Physical Diagnostics and Dental Physiotherapy, Pomeranian Medical University in Szczecin, al. Powstańców Wielkopolskich 72, 70-111 Szczecin, Poland; 5 Department of Genetics and Pathology, Pomeranian Medical University in Szczecin, ul. Unii Lubelskiej 1, 71-252 Szczecin, Poland

**Keywords:** Radiotherapy, 3D printed boluses, Radiation-induced physicochemical changes, Engineering, Materials science, Physics

## Abstract

In radiotherapy, boluses ensure dose buildup at the skin and adapt to complex anatomy, requiring stable, well-conforming materials. This study aimed to investigate the effect of ionising radiation (70 Gy, 6 MV) on changes in the physicochemical properties of two materials used for 3D printing bolus materials for radiotherapy: acrylonitrile butadiene styrene ABS and thermoplastic copolyester TPC. Surface roughness, tribological parameters, hardness, dimensional stability, Fourier transform infrared spectra FTIR, and differential scanning calorimetry DSC data were assessed. After irradiation, small statistically significant changes in roughness parameters were observed for both materials, with ABS exhibiting greater surface degradation. In tribological tests, ABS demonstrated a 70% reduction in coefficient of friction, while TPC remained stable. FTIR spectra revealed changes characteristic of ABS degradation and oxidation (decreases in the intensity of butadiene bands, CH₂ shifts, and increases in carbonyl bands). Subtle chemical stability and potential cross-linking were observed in TPC. DSC suggests no changes in TPC and a decrease in the glass transition temperature of ABS, suggesting slight structural degradation. A slight increase in hardness was observed in ABS and TPC after irradiation, with only minimal dimensional changes in ABS. Based on the data obtained, X-ray radiation affected the tested materials differently. Although both retain their function after exposure to therapeutic doses, TPC exhibits greater chemical and mechanical resistance. Combined with its greater flexibility, this may result in improved clinical adhesion and reproducibility during radiotherapy. Further studies will include analysis of adhesion and dose distribution (Part 2).

**Supplementary Information:**

The online version contains supplementary material available at 10.1038/s41598-026-36952-x.

## Introduction

In radiotherapy, one key element of planning is ensuring adequate irradiation of superficial tissues^1–3^. When using high-energy photon beams, the maximum radiation dose occurs several millimetres below the skin surface, reducing the dose in the epidermis and dermis. This phenomenon, known as the built-up effect, is beneficial in protecting healthy tissues; however, in superficial lesions, it can lead to insufficient irradiation^1,4–6^.

A bolus is a material with properties similar to soft tissue, placed on the patient’s body surface to shift the maximum dose point toward the skin surface. It is also used to compensate for anatomical irregularities and reduce air gaps between the skin surface and the radiation beam, improving dose uniformity^6–9^. For example, in the head and neck region, due to the patient’s complex anatomy, the bolus plays a vital role in ensuring precise dose delivery to the target area and protecting critical structures through appropriate dose distribution modelling^9–11^. Traditionally, boluses are made of flexible materials, such as paraffin, which are manually modelled on the patient, a process that can be time-consuming and less reproducible^7,8,12^.

Over the past decade, 3D printing technology has been used in oncology, including radiotherapy, as a tool for personalising components used in treatment planning. One area where 3D printing has provided significant added value is the production of boluses tailored to the patient’s anatomy. This allows for a more precise fit to the patient’s surface, reduced air gaps, and increased positioning consistency during subsequent treatment fractions. This solution can improve the consistency of the treatment plan with the actual dose distribution delivered during irradiation and shorten patient preparation time^4,7–9^.

The development of this technology has also enabled a significant reduction in the time required to prepare the patient for treatment, as well as increased patient comfort through improved bolus adjustment to the patient’s anatomy^2,7,9,10^. The literature also describes examples of integrating 3D printing with other radiotherapy planning tools, such as dose distribution simulation software, which enables additional verification of bolus quality before its execution^6,8,10^.

As 3D printing is an increasingly popular technology offering a wide range of materials, its stability during therapeutic administration is essential. Changes in mechanical, chemical, or geometric properties can impact the clinical efficacy and safety of therapy. Therefore, assessing the radiation resistance of materials used to print boluses is an essential element of research into their clinical utility.

This study aims to assess the effects of therapeutic X-ray doses on the physical, chemical, and thermal properties of two thermoplastic materials used for 3D printing bolus implants in radiotherapy: acrylonitrile butadiene styrene (ABS) and a copolyester thermoplastic (TPC). To date, the stability of bolus implants after exposure to therapeutic radiation has been analysed sporadically, most often concerning single filaments. There are a few reports directly comparing different materials in terms of changes in their properties after irradiation, which could affect the efficacy and repeatability of treatment^5,13^. Such studies are of practical importance for selecting optimal materials to ensure the durability and safety of bolus implants in radiotherapy. We chose ABS and TPC for their practical significance in the production of radiotherapy boluses via 3D printing. ABS is the standard material used in Poland for the production of individual boluses, making it a natural reference point. TPC, on the other hand, was chosen because, among the materials compatible with our 3D printers, it was the most flexible, making it a promising candidate for adapting to the patient’s surface. These considerations justified evaluating both polymers for radiotherapy applications.

## Materials and methods

### Materials and sample preparation

ABS and TPC are thermoplastic polymers commonly used in 3D printing, as they can be moulded after heat treatment. ABS is characterised by high stiffness and dimensional stability, while TPC is a flexible material with greater fracture resistance and the ability to conform to complex surfaces. The mechanical properties of both materials, as declared by the producer, are presented in Supplementary Table S1. The printing parameters used in the study are summarised in Supplementary Table S2. The study used ABS filament (Zortrax, Poland) and TPC filament (PRINT-ME Flex, Poland), both with a diameter of 1.75 mm and standard technical parameters intended for FDM printing.

The test samples were designed in Fusion 360. Supplementary Fig. S1 presents sample models prepared for testing, showing three variants of 3D print dimensions. The file was exported to the stereolithography format, which is compatible with the 3D printer’s software, and then printed on a Zortra M300 Dual printer using FDM technology.

For the study, cuboid samples of ABS and TPC filaments were prepared, varying in size depending on the planned measurement method:

- Measurements of dimensional changes after irradiation and analysis of tribological parameters were performed on samples measuring 20 × 20 × 5 mm^3^, n = 5 for each material
- Surface roughness measurements, Shore D hardness, and surface chemical composition analysis were performed on samples measuring 10 × 10 × 5 mm^3^, n = 5 for each material
- Samples measuring 4 × 4 × 1 mm^3^ were prepared for analysis of thermal properties, n = 1 for each material

The designation “K” is used to mark the control (non-irradiated) samples; sample codes without the “K” refer to irradiated specimens.

### Dimensional change measurements

Accurate dimensional measurements of the bolus are essential. Even minor deviations in thickness can significantly affect the surface dose distribution during radiotherapy. The dimensions (x) of all samples were measured five times each using an electronic calliper with an accuracy of 0.02 mm and a resolution of 0.01 mm, both before and after irradiation.

### Tribological property analysis

The assessment of tribological properties provides insight into the surface wear resistance and frictional behaviour of bolus materials. These parameters are essential for evaluating bolus durability during repeated handling and positioning during radiotherapy sessions. To assess the tribological properties of the materials (ABS and TPC), a tribometer, TRB3, designed for use in both dry and liquid environments, was used from Anton Paar. Tribological experiments were conducted at room temperature, with a relative humidity of 40 ± 2%, and without lubricants. The friction pair in contact during the tribological experiment consisted of a ceramic ball (Al2O3) with a diameter of 6.0 mm, which was fixed in a holder above the table and prevented from rotating. The evaluated sample of polymer material (ABS and TPC) was mounted in the holder and rotated, resulting in sliding friction between the materials. The friction coefficients and wear rate were measured for irradiated and non-irradiated samples. The radius of the circle along which the friction pair moved (distance from the centre of the examined sample) was 7 mm. The sample was rotated on a rotating table at 100 revolutions per minute under an applied load (FN) of 5 N for 40 min; the travelled path length was 150 m. Tribological experiments were performed according to the ASTM G99-95 standard^14^.

Damage after tribological measurement was assessed using a SENSOFAR S Neox confocal microscope with a 5 × objective magnification. After the tribological test, tribological traces remain on the tested samples’ surfaces due to the counterbody’s influence. Using a microscope, the transverse profile at four locations with 90-degree intervals and the cross-sectional area were also established, along with circular grinding traces at each location according to the ČSN EN 1071-13:2010 standard^14^.

### Surface roughness measurements

Surface roughness affects the contact quality between the bolus and the patient’s skin, influencing the presence of air gaps and, consequently, the accuracy of dose delivery. Precise roughness analysis enables optimisation of the manufacturing process to improve clinical performance. The surface roughness was evaluated using a non-contact optical 3D profilometer, S Neox from SENSOFAR, with a 10 × objective, according to ISO 25,178^15^. The data were averaged from three measurements in different areas of the sample surface for every five samples. The average arithmetic height of the surface (Sa), the kurtosis (Sku), the skewness (Ssk), the maximum pit depth (Sv), the maximum peak height (Sp), the root mean square height (Sq), and the maximum height of the surface (Sz) were monitored. Measurements were performed 3 times for each sample group, before and after irradiation.

### Shore D hardness measurements

Hardness is particularly relevant for boluses, as it relates to their ability to maintain shape and thickness during patient positioning. For hardness measurements, based on the filaments manufacturer’s data, the Shore hardness gauge durometer LD0551 from TQC, with an accuracy of 0.006, was selected. Five hardness measurements were performed for each of the five samples, before and after irradiation.

### Surface chemical composition analysis

Fourier-transform infrared spectroscopy (FTIR) enables the identification of chemical functional groups in the material and the detection of structural changes. Monitoring these after irradiation is vital for assessing the long-term stability of bolus materials. FTIR was conducted on the Nicolet iS10 (Thermo Scientific, USA) spectrometer using a single-reflection attenuated total refectance (ATR) system equipped with a diamond crystal. The samples were used as received, before and after radiation exposure. Each infrared (IR) spectrum consisted of 32 scans over 400–4000 cm^−1^ with a resolution of 4 cm^−1^.

### Thermal property analysis

Differential scanning calorimetry (DSC) is one of the most commonly applied techniques in thermal analysis. It measures changes in the enthalpy of a sample associated with variations in its physical or chemical properties as a function of temperature or time^16,17^. DSC was conducted on a DSC 1/700 calorimeter (Mettler Toledo, Switzerland) to study thermal properties before and after radiation exposure. Samples for measurements were prepared using an RM 2255 microtome from Leica (Wetzlar, Germany) from the middle part of specimen cross-sections (6 ± 0.5 mg). DSC analysis was performed under a nitrogen flow rate of 50 mL min^−1^, with the first heating scan conducted from 0 to 250 °C at a heating rate of 10 °C min^−1^.

### Irradiation procedure

After measurements, all prints were irradiated with a single dose of 70 Gy using a Varian Edge linear accelerator operating at 6 MV. All measurements were repeated for all irradiated samples. The dose of 70 Gy was chosen as representative of the typical total therapeutic dose used in radical radiotherapy regimens (e.g., head and neck cancers), thereby enabling the assessment of changes in material properties under conditions that correspond to real clinical practice.

### Statistics

The data distribution was assessed using the Shapiro–Wilk test. For normally distributed data, the statistical significance of group differences was evaluated using Student’s t-test. For non-normally distributed data or when there are too few measurements, the Mann–Whitney U test was used (Statistica 13). Differences were considered statistically significant at *p* < 0.05.

## Results

### Dimension

Results of dimension measurements are presented in Supplementary Table S3. The results of the data distribution analysis are included in Supplementary Table S4. As the data in Supplementary Table S5 show, only for ABS is a slight (0.05 mm) statistically significant change in dimension observed. No statistically significant change in dimensions was observed for TPC.

### Tribological property analysis

**Fig. 1:**
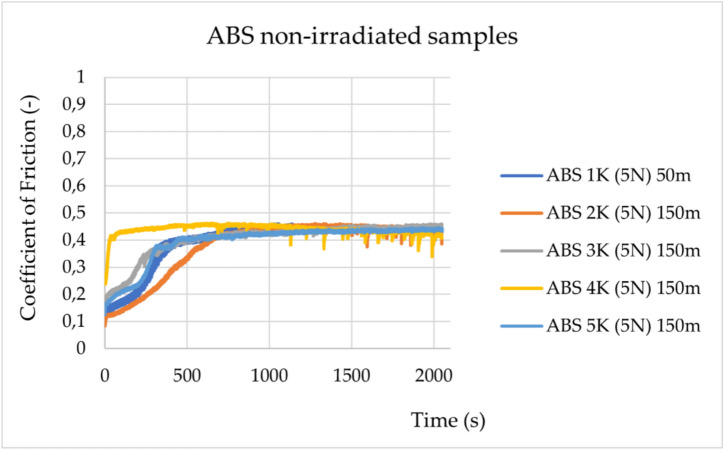
Change in friction coefficients during experiments for the non-irradiated ABS samples (ABS 1 K-ABS 5 K) under a load of 5 N. The sliding distance was 50 m for the ABS 1 K sample and 150 m for the ABS 2 K-5 K samples.

**Fig. 2:**
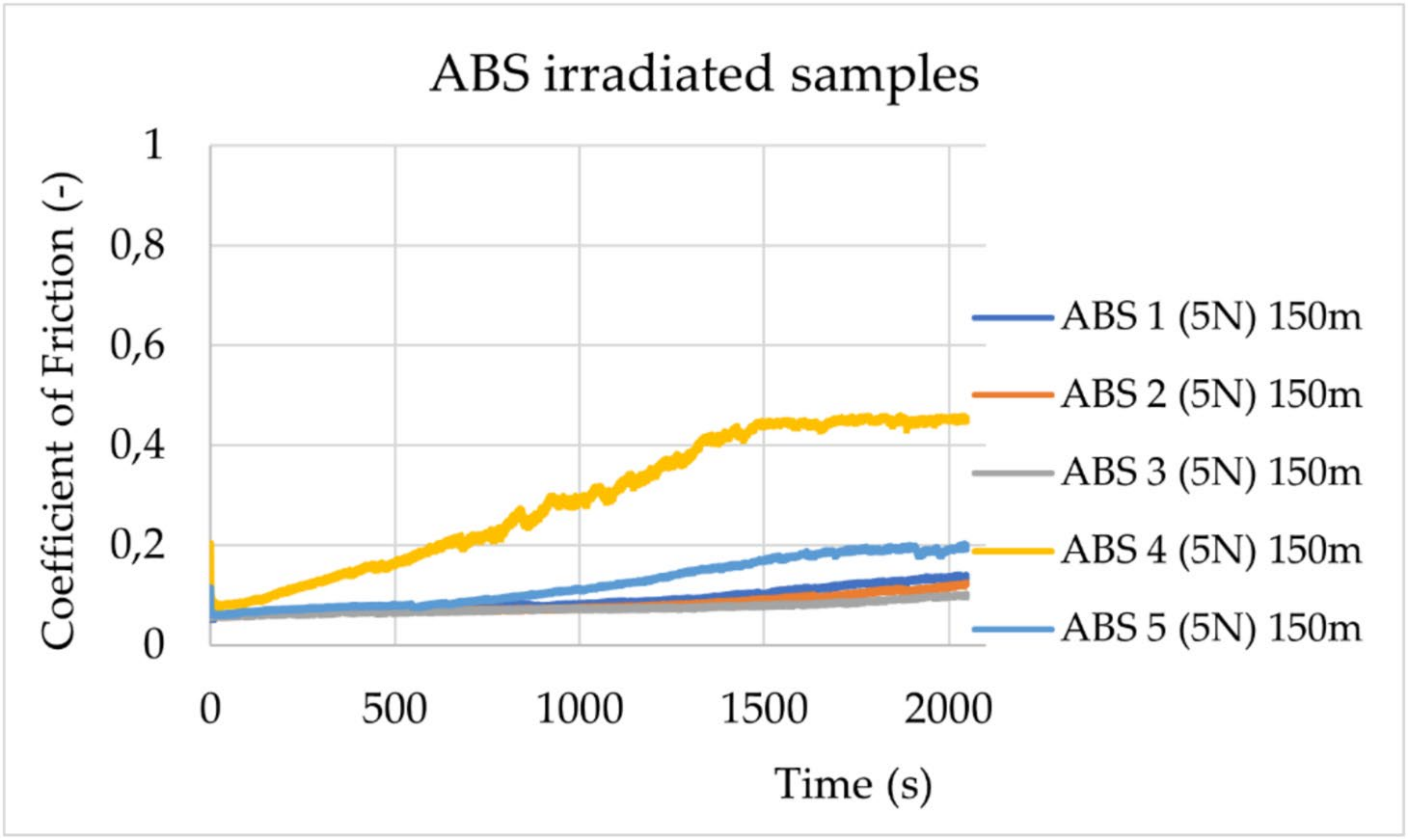
Change in friction coefficients during experiments for the irradiated ABS samples (ABS 1-ABS 5) under a load of 5 N. The sliding distance was 150 m.

Friction properties were observed for irradiated and non-irradiated ABS and TPC samples. It can be observed that ABS exhibits a change in tribological behaviour after irradiation. The change is observed in a decrease in the friction coefficient value by approximately 70%. In contrast, TPC shows only minimal changes in tribological behaviour after irradiation. The change in friction coefficients during the group’s experiments on ABS is shown in Figs. 1 and 2.

**Fig. 3:**
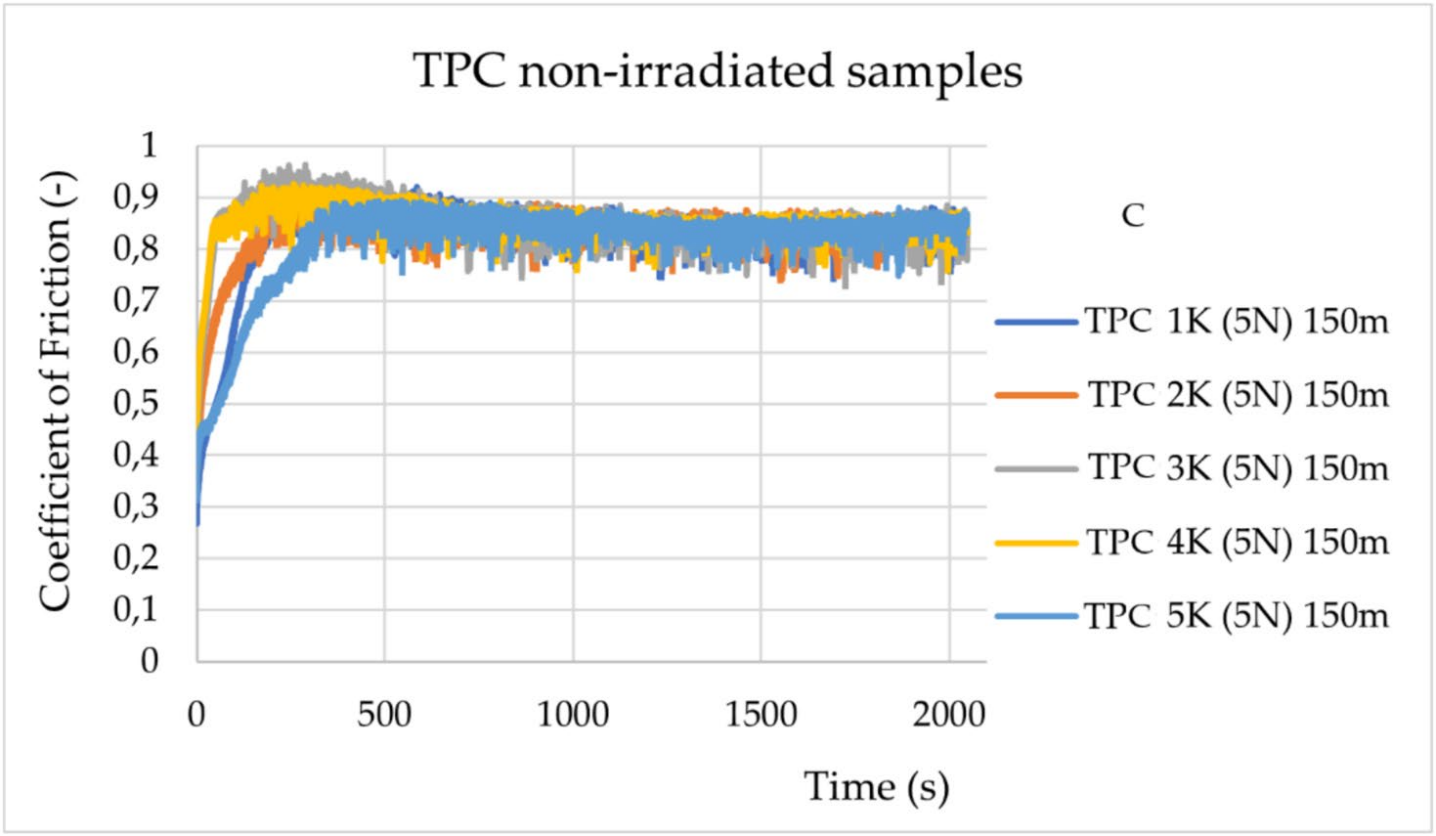
Change in friction coefficients during experiments for the non-irradiated TPC samples (TPC 1 K- TPC K5) under a load of 5 N. The sliding distance was 150 m.

**Fig. 4:**
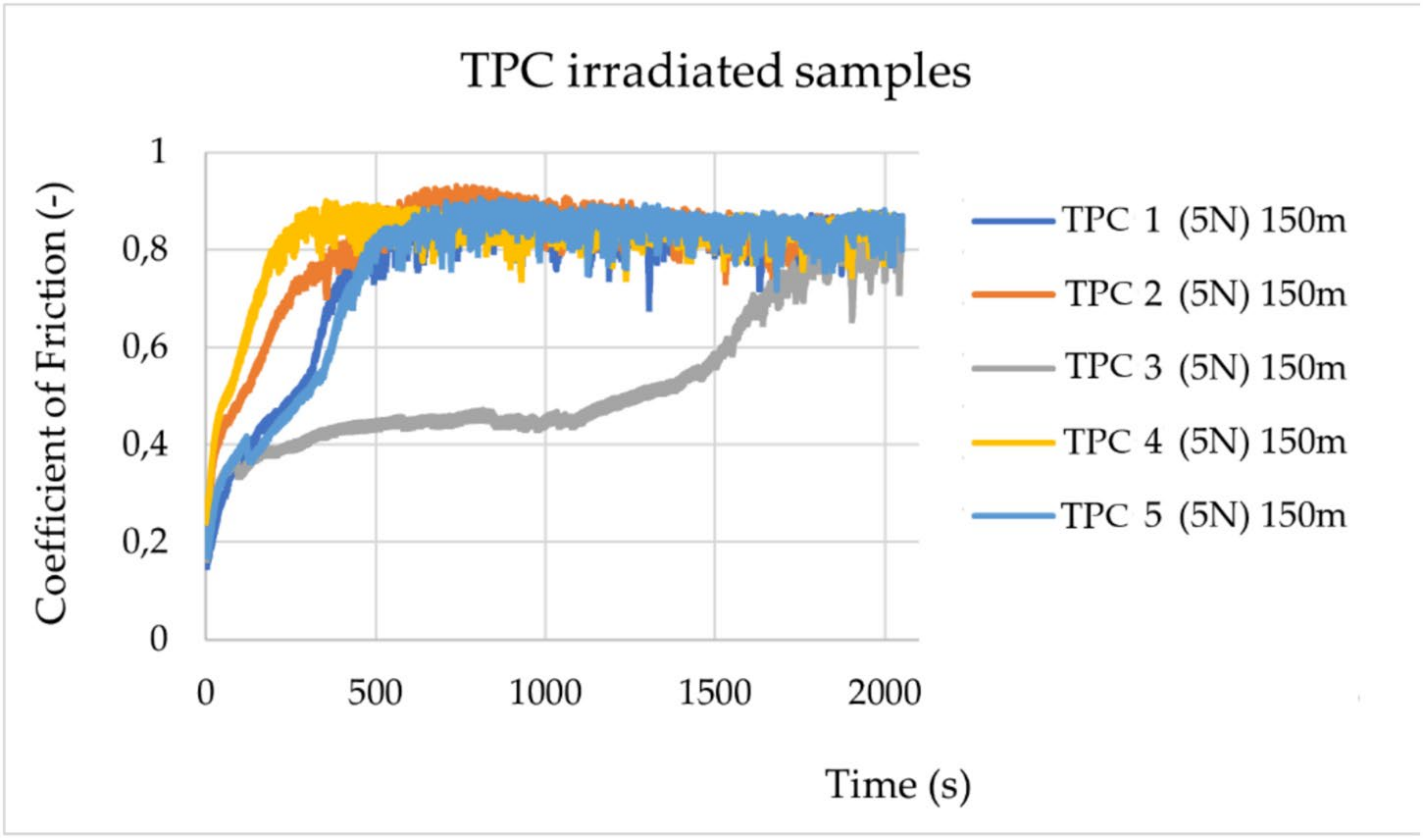
Change in friction coefficients during experiments for the irradiated TPC samples (TPC 1- TPC K) under a load of 5 N. The sliding distance was 150 m.

In the TPC samples, significant surface damage occurred upon contact with the counter body (a ball made of Al2O3) (Fig. 3). The change in the samples’ friction properties was recorded only at the beginning of the measurement, as shown in the graphs in Fig. 4, which indicate a shift in the zone with a lower friction coefficient, compared to those in Fig. 3. This type of material shows statistically significant, but minimal, changes in friction behaviour, with a magnitude much lower than in ABS.

**Fig. 5:**
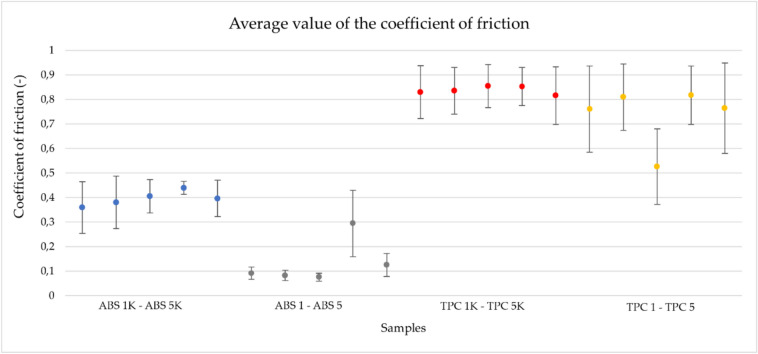
Comparison of average values of friction coefficients for the monitored groups of samples, with error bars representing standard deviation. Blue points—non-irradiated ABS samples (ABS 1 K ABS 5K0, grey—irradiated ABS samples (ABS 1–ABS 5), red—non-irradiated TPC samples (TPC 1 K–TPC 5 K), yellow—irradiated TPC samples (TPC 1—TPC 5).

Figure 5 compares the average friction coefficients and their corresponding standard deviations. Again, it can be stated that the ABS material is more affected by irradiation than the TPC material.

The statistical data and their distribution analysis are presented in Supplementary Tables S6 and S7. As shown in Supplementary Table S8, for both materials, ABS and TPC, a statistically significant decrease in the friction coefficient values is observed after irradiation. However, the change in value is greater for ABS (0.305) than for TPC (0.071).

It was challenging to evaluate the wear rate of the samples because their surface roughness was too high. For samples from the ABS, wear can only be assessed visually by comparing the surfaces on which damage is visible before and after irradiation, with the surface remaining almost undamaged (supplementary Figs. S2 and S3 present samples after tribological evaluation).

Supplementary Table S9 contains statistical data on the width and depth of the wear rate for TPC samples. Due to the small sample size, a nonparametric test was used to compare the groups. The results, presented in Supplementary Table S10, indicate that there are no statistically significant differences between the unexposed and exposed samples. An example of confocal microscopy analysis of a wear track after tribological testing is shown in Supplementary Fig. S4. To better understand whether the observed changes in friction are related to surface morphology, roughness analysis was performed.

### Surface roughness measurements

An example of surface topography analysis performed using confocal microscopy is presented in Supplementary Fig. S5. Statistical data on roughness parameters and their distribution analysis are presented in Table 1. Data distribution analysis is presented in Supplementary Tables S11 and S12. The results of the comparison between exposed and unexposed samples, presented in Supplementary Table S13, show statistically significant decreases in all evaluated parameters, except Sa for ABS, Sku for TPC, and Ssk for both materials. Slightly larger changes are observed in the case of ABS.

**Table 1:** Statistical data for the surface roughness parameters for ABS and TPC polymer samples. Sa, the average arithmetic height of the surface; Sku, the kurtosis; Ssk,  the skewness; Sv, the maximum pit depth; Sp, the maximum peak height; Sq, the root mean square height; Sz, the maximum height of the surface.

parameter	Radiation	Mean	Median	Minimum	Maximum	25th Percentile	75th Percentile	Standard deviation
ABS								
Sa	Before	13.90	13.94	12.27	15.94	13.27	14.56	0.91
	After	13.25	13.47	11.41	15.41	12.28	14.01	1.18
Sku	Before	8.70	8.53	6.02	14.11	6.73	10.46	2.24
	After	5.06	4.45	3.37	8.55	4.05	5.84	1.56
Sp	Before	144.71	141.86	113.51	216.95	123.96	148.96	26.93
	After	105.72	111.45	83.21	131.34	90.61	116.79	15.26
Sq	Before	18.77	18.53	16.19	21.51	17.73	19.97	1.54
	After	17.15	17.40	15.02	21.26	15.35	17.87	1.78
Ssk	Before	0.46	0.38	0.01	1.42	0.15	0.62	0.41
	After	0.28	0.19	0.01	0.85	0.09	0.46	0.26
Sv	Before	149.01	154.70	92.07	206.06	118.75	183.05	34.95
	After	108.90	110.23	82.31	135.59	90.64	123.76	16.42
Sz	Before	293.72	304.04	215.34	395.17	250.91	323.68	47.18
	After	214.62	215.77	167.58	258.26	194.21	240.43	27.61
TPC								
Sa	Before	13.41	12.64	8.522	19.66	9.77	16.94	3.77
	After	10.51	9.38	7.38	17.62	8.31	12.95	2.94
Sku	Before	7.27	7.77	4.60	9.93	5.89	8.39	1.52
	After	6.40	6.17	4.02	9.73	5.15	7.60	1.66
Sp	Before	139.91	140.64	91.73	183.89	131.13	153.57	23.54
	After	109.30	110.39	79.71	135.63	98.89	128.54	18.28
Sq	Before	18.61	18.46	11.36	28.68	13.30	24.05	5.69
	After	14.05	12.13	9.19	23.96	10.69	18.73	4.34
Ssk	Before	1.05	1.01	0.34	1.84	0.72	1.33	0.46
	After	0.90	0.83	0.01	2.02	0.59	1.22	0.50
Sv	Before	123.75	120.06	83.03	178.27	98.15	151.15	27.28
	After	102.69	96.83	79.94	141.62	84.34	116.25	20.13
Sz	Before	263.65	266.36	174.76	322.82	238.79	286.59	41.38
	After	211.98	211.77	171.66	272.65	191.42	236.77	29.02

### Shore D hardness measurements

Statistical data on Shore D hardness for both materials, before and after exposure, and an analysis of their distribution are presented in Supplementary Tables S14 and S15, respectively. As shown in Supplementary Table S16, small statistically significant increases in hardness of 3 were observed for both polymers after irradiation. To investigate whether the surface modifications correspond to underlying chemical changes, FTIR analysis was conducted.

### Surface chemical composition analysis

FTIR analysis was used to identify qualitative trends associated with radiation-induced degradation mechanisms rather than to provide quantitative analysis of oxidation products. The characteristic FTIR bands corresponding to different types of butadiene units in ABS were identified as follows: vinyl-butadiene shows peaks at approximately 990 cm^−1^ and 910 cm^−1^, corresponding to C = C–H out-of-plane bending vibrations; trans-butadiene exhibits a band near 967 cm^−1^, characteristic of trans-1,4-polybutadiene units; while an alternative vinyl-butadiene band appears around 1640 cm^−1^, which is often preferred for mapping due to reduced overlap with neighbouring peaks. Chain scission in ABS after X-ray irradiation can be detected by monitoring changes in characteristic absorption bands associated with the polymer’s chemical structure. Specifically, FTIR can reveal:

1. a decrease in the intensity of bands associated with butadiene units (such as vinyl and trans-butadiene peaks), indicating breaking of polymer chains in the butadiene phase, which is more susceptible to degradation;
2. an increase in carbonyl (C =O) and hydroxyl (OH) bands near the surface, which are products of oxidation resulting from chain scission and subsequent reactions with oxygen;
3. shifts in CH2 stretching bands (e.g., red shifts from 2848 to 2853 cm^−1^ and from 2922 to 2925 cm^−1^) that suggest disorganisation of polymer chains and increased chain mobility due to chain scission.

No decrease was observed in the intensity of the vinyl-butadiene (1,2-polybutadiene) peaks at approximately 990 cm^−1^ and 910 cm^−1^, which correspond to C=C–H out-of-plane bending vibrations characteristic of vinyl groups (see Supplementary Fig. S6).

As shown in Supplementary Fig. S7, no decrease was observed in the trans-butadiene (trans-1,4-polybutadiene) peak around 967 cm^−1^, which is assigned to the trans-1,4-polybutadiene units in the polymer. Additionally, the vinyl-butadiene units can also be mapped using a band near 1640 cm^−1^, which is a better alternative for vinyl-butadiene distribution than the 911 cm^−1^ band because the latter overlaps with polystyrene absorption. Supplementary In Fig. S8, a slight decrease of the 1640 cm^−1^ band after irradiation might indicate breaking of polymer chains in the butadiene phase.

Carbonyl (C = O) bands: typically appear in the range 1800–1600 cm^−1^, with a common peak around 1720–1743 cm^−1^ depending on the specific carbonyl species formed (e.g., ketones, aldehydes, acids). A decrease in ester (1740 cm^−1^), ketone (1700 cm^−1^), and aldehyde (1730 cm^−1^) groups after irradiation can indicate crosslinking in the polymer. Supplementary Fig. S9 shows a decrease in intensity after irradiation, suggesting that these oxidised fragments are being consumed or transformed through crosslinking reactions, in which polymer chains form covalent bonds linking them together.

Hydroxyl (OH) bands: Appear broadly in the range 3600–3200 cm^−1^, corresponding to O–H stretching vibrations from alcohols, hydroperoxides, or carboxylic acids formed during oxidation. Supplementary Fig. S10 shows slight consumption or transformation of hydroxyl groups. Some OH groups may be chemically transformed or consumed during irradiation-induced reactions such as crosslinking or oxidation, leading to fewer detectable hydroxyls.

Typical FTIR peaks observed in TPC thermoplastic elastomers correspond to the characteristic functional groups of polyester-based polymers. These include:

1. 1750–1650 cm^−1^: Strong peak due to the carbonyl (C=O) stretching vibration of ester groups, a key signature of the polyester segments in TPC elastomers;
2. 3000–2900 cm^−1^: Peaks attributed to C–H stretching vibrations from aliphatic methyl (CH3) and methylene (CH2) groups;
3. 1500–1200 cm^−1^: Peaks mainly from C–H bending (deformation) modes and C–O stretching vibrations in ester and ether groups;
4. 1100 cm^−1^ region: Characteristic of C–O stretching in ester and ether linkages;
5. 1000–600 cm^−1^: Complex vibrations involving C–O–C ether and ester bonds, C–C skeletal vibrations, and out-of-plane bending modes.

Hydroxyl (OH) bands: Appear broadly in the range 3600–3200 cm^−1^, corresponding to O–H stretching vibrations from alcohols, hydroperoxides, or carboxylic acids formed during oxidation. Consumption or transformation of hydroxyl groups has been observed. Some OH groups may be chemically transformed or consumed during irradiation-induced reactions such as crosslinking or oxidation, leading to fewer detectable hydroxyls. The absorbance peaks in the 3000–2900 cm^−1^ range are related to stretching vibrations of aliphatic C-H bonds found in methyl (CH3) and methylene (CH2) groups. A shift from 2937.2 cm^−1^ to 2917.3 cm^−1^ reflects degradation and structural changes, such as chain scission, oxidation, or cross-linking, which affect the vibrational characteristics of aliphatic C-H bonds and indicate material ageing or modification due to radiation exposure.

The FTIR spectra of TPC before and after irradiation are presented in Supplementary Fig. S11. The results show characteristic C–H stretching bands in the 3000–2800 cm^−1^ region and a broad O–H band around 3400 cm^−1^. After irradiation, a decrease in the intensity of the hydroxyl band and subtle variations in the C–H stretching region were observed, suggesting minor structural rearrangements and oxidation effects.

A sharp peak at 1715 cm^-1^ typically corresponds to the carbonyl (C = O) stretching vibrations. This region is characteristic of ester groups present in the polyester segments of the TPC copolymer. Supplementary Fig. S12 shows that no changes have been observed after irradiation.

The 1500–1200 cm^−1^ range includes C–H bending vibrations (scissoring, wagging) from aliphatic groups. It also covers C–O stretching vibrations from ester linkages and ether groups present in the polyester segments of TPC elastomers. Small changes in this region can indicate modifications in the polymer structure, such as chain scission, cross-linking, or oxidation after irradiation. FTIR peak around 1100 cm^−1^ in a TPC thermoplastic elastomer is generally attributed to C–O stretching vibrations in ester groups and possibly also to C–O–C ether linkages present in the polymer backbone. This region is characteristic of the stretching vibrations of carbon–oxygen single bonds in esters, ethers, and related functional groups, which are common in polyester-based elastomers. X-ray irradiation may promote oxidation or other chemical reactions that increase the number of C–O-containing groups (e.g., the formation of new ester, ether, or hydroxyl groups), leading to a stronger C–O stretching absorption peak due to radiation-induced oxidation or chemical modification. The 1000–600 cm^−1^ FTIR peaks in TPC thermoplastic elastomers are associated with C–O and C–C vibrations in ester and ether groups and provide detailed information about the polymer’s backbone structure and any chemical or physical changes it undergoes. X-ray radiation may disrupt the polymer’s ordered structure, affecting vibrational modes and thus decreasing peak intensity in this region, as shown in Supplementary Fig. S13.

FTIR results show that irradiation causes typical degradation in ABS, such as oxidation (carbonyl group formation) and chain scission (fewer butadiene bands and CH₂ shifts). In contrast, TPC shows only subtle spectral changes and appears more chemically stable, with possible radiation-induced crosslinking rather than noticeable chain scission. These FTIR findings align with observed differences in surface, thermal, and mechanical behaviour between the materials.

### Thermal property analysis

**Fig. 6:**
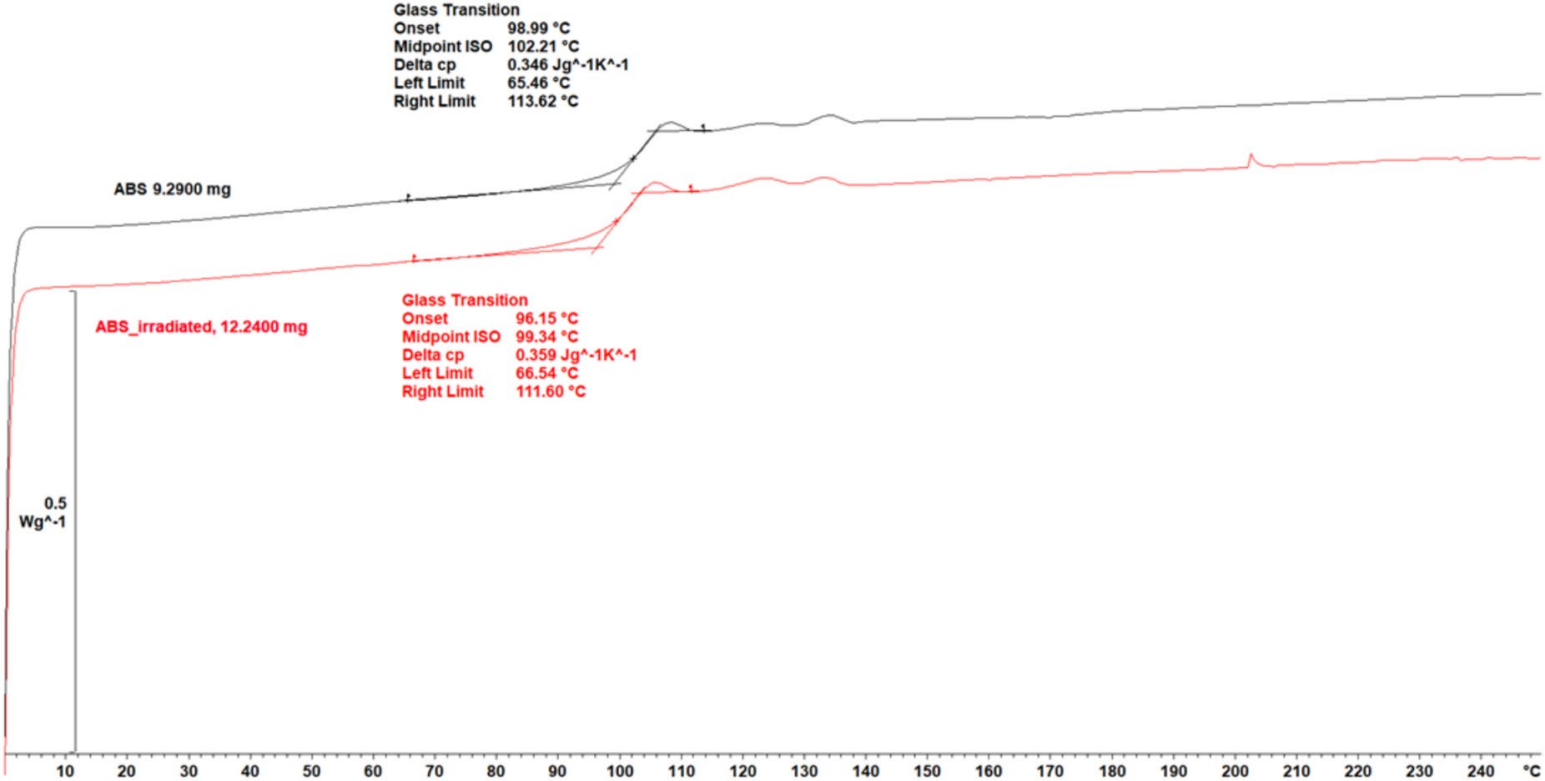
DSC analysis results for ABS before (black) and after (red) irradiation. A shift in the glass transition temperature (Tg) is observed—the non-irradiated sample showed an onset at 98.9 °C and a midpoint at 102.2 °C, while the irradiated sample presented an onset at 96.1 °C and a midpoint at 99.3 °C, respectively.

Finally, DSC measurements were used to assess whether irradiation affected the thermal stability of the polymer backbone. A decrease in Tg from 102.2 to 99.3 °C after X-ray irradiation, as shown in Fig. 6, suggests that the ABS has undergone slight structural degradation (chain scission), leading to increased chain mobility and potentially reduced thermal stability.

**Fig. 7:**
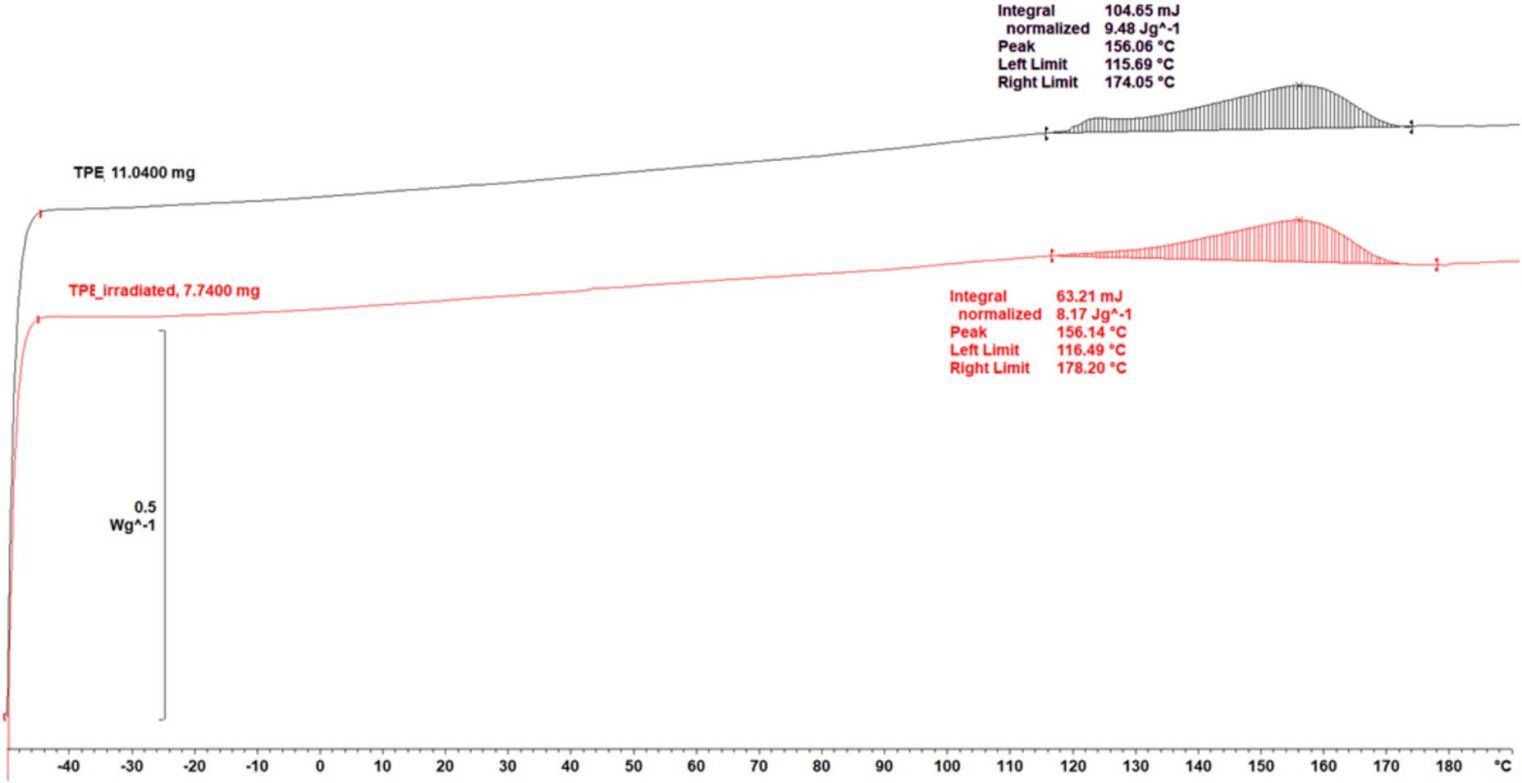
DSC analysis results for TPC before (black) and after (red) irradiation. In both cases, an endothermic peak was detected at a similar temperature.

In Fig. 7, no changes in melting behaviour and thermal characteristics of TPC after X-ray irradiation suggest that the polymer is stable after exposure.

## Discussion

ABS, which can be considered a standard in 3D bolus printing, is a well-researched material used clinically in many centres. However, its main drawbacks are stiffness and limited deformability, which, in complex anatomical shapes, can lead to air gaps and disturbances in dose distribution. Therefore, interest in more flexible materials, such as TPU thermoplastic polyurethane, is a natural development direction. TPU, due to its high deformability, has been the subject of numerous studies^5,9,18–20^. Still, it is characterised by difficulties in the printing process and high sensitivity to moisture and temperature. The technical and materials literature emphasises that TPU, despite its high flexibility, has significant practical limitations. It is highly hygroscopic, absorbing moisture from the environment, which can lead to reduced print quality and mechanical property degradation if the filament is not properly dried and stored^21–23^. There are no reports in the literature on the use of TPC in radiotherapy, making our research pioneering. TPC can be considered a compromise between rigid ABS and highly flexible TPU – it is more susceptible to deformation than ABS, but also easier to print and more environmentally stable than TPU^24,25^. This may be particularly important when designing boluses, for example, for the head and neck region, where a balance between flexibility for precise adhesion and stiffness for shape stability during multi-fraction therapy is required. Compared to more commonly studied bolus materials, such as TPU, PLA, and silicone composites, TPC appears to occupy a middle ground. TPU is highly deformable but highly hygroscopic and sensitive to processing conditions, which significantly complicate printing and potentially reduce mechanical stability. PLA is easy to print but has limited thermal resistance and is susceptible to radiation-induced changes. Silicones offer excellent conformability to the skin surface but do not provide the same level of shape precision and personalisation as 3D-printed boluses. In this context, TPC may represent a compromise material—combining favourable flexibility with greater environmental stability and a more straightforward printing process than TPU, increasing its potential for bolus applications in radiotherapy. In clinical use, TPU’s tendency to soften at relatively low temperatures may compromise its shape stability when placed on warm, perspiring skin, potentially causing deformation, reduced reproducibility between fractions and an increased likelihood of air-gap formation.

The obtained results indicate that both ABS and TPC undergo small, though statistically significant, changes in dimensions and hardness after irradiation. The increase in hardness may be due to radiation-induced cross-linking. In clinical practice, increased hardness may enhance the mechanical stability of the bolus; however, excessive stiffness limits its ability to conform precisely to the patient’s skin. This may lead to the formation of small air gaps, decreased patient comfort and reduced precision of dose delivery, particularly in regions where close surface adaptation is essential. This aspect requires consideration, where precise reproduction of anatomical surfaces is crucial.

The tribological analysis showed a significant decrease in the coefficient of friction for ABS, attributed to surface smoothing, chemical changes, and increased hardness. Theoretically, a reduction in friction could have both positive (easier positioning) and negative (increased risk of slippage) effects. Still, a complete evaluation requires tests planned for the next part of the project (Part II), including analysis of the bolus’ adhesion and behaviour under clinically relevant conditions. In the case of TPC, the changes were minimal, suggesting that this material maintains greater surface stability with repeated use.

Similarly, roughness analysis revealed significant decreases in the Sa, Sv, and Sz parameters for both materials, with the changes being more pronounced for ABS. Surface smoothing can reduce air gaps and improve bolus adhesion to the skin. However, in the case of ABS, this effect coincided with signs of chemical degradation, which may compromise the material’s long-term stability.

The observed surface smoothing can be attributed to chemical processes suggested by FTIR and DSC. The decreased intensity of the butadiene bands, along with the appearance and modification of the carbonyl bands, indicates chain rupture and oxidation in the ABS surface layer. At the same time, the decrease in Tg observed by DSC suggests increased mobility of polymer segments after irradiation. The combination of these phenomena may lead to local reorganisation of the material and a reduction in the sharpness of microcracks and topographic peaks, resulting in a smoother surface. It should be noted that FTIR results are interpreted qualitatively and are intended to support the identification of dominant degradation mechanisms rather than to provide quantitative chemical analysis.

FTIR analysis suggests a greater susceptibility of ABS to radiation-induced degradation, as evidenced by decreased butadiene band intensity, the appearance of carbonyl bands and shifts in the CH₂ bands. In contrast, spectral changes observed for TPC were subtle and limited mainly to minor shifts in the C–H and C–O bands, indicating higher resistance to chain scission and possible radiation-induced crosslinking. Consistently, DSC revealed a decrease in the glass transition temperature of ABS by approximately 3 °C, while TPC retained thermal stability. The Tg reduction in ABS suggests a slight increase in polymer chain mobility induced by irradiation. Although this shift is subtle and the Tg of ABS remains well above typical skin and ambient temperatures encountered during radiotherapy, increased chain mobility may influence material behaviour during repeated clinical use, contributing to gradual surface or mechanical changes such as altered friction or stiffness. In terms of long-term storage, the observed Tg decrease does not indicate immediate loss of functionality. Still, it may reflect early-stage structural degradation that could accelerate ageing processes under unfavourable environmental conditions.

Our results are consistent with previous reports on ABS. In Jezierska et al.^11^, 60 Gy caused no significant changes in hardness and only 0.3–0.4% dimensional shrinkage (< 0.1 mm). In our work, 70 Gy produced similarly small dimensional changes (0.05 mm; ~ 0.25%), a modest ~ 3 °C decrease in Tg, and limited FTIR signs of oxidation and chain scission. Jreije et al.^26^ reported moderate mechanical degradation of ABS composites after repeated recycling, with minimal additional deterioration after 70 Gy. Şahin et al.^27^ also documented only limited thermo-mechanical changes in ABS at radiotherapy-relevant conditions. Most importantly, Şahin et al.^13^ showed radiation-induced reductions in Sa, Sz and Sv, as well as altered friction and wear behaviour, with magnitudes comparable to those observed in our study. These findings suggest that the physicochemical and surface modifications detected here fall within known ranges of radiation-induced changes in ABS.

The lack of reports on TPC, however, emphasises the innovative nature of this work. Furthermore, TPU is characterised by lower thermal resistance, softens at elevated temperatures, and has a lower melting point than TPC^25,28^. In comparison, ABS, according to the results of Lendvai et al.^23^, is significantly less susceptible to humidity, making it a more predictable material in printing. In this context, TPC may represent an attractive compromise: it is less sensitive to moisture and temperature than TPU, while being easier to print and more flexible than ABS, thereby increasing its potential for bolus applications in radiotherapy.

The study had certain limitations. The relatively small sample sizes (n = 5 for some tests and n = 1 for DSC) limit the study’s statistical power; the thermal results, in particular, should be interpreted as qualitative trends rather than precise population estimates. Only a single irradiation dose of 70 Gy was studied, whereas in clinical settings, the bolus is repeatedly exposed during subsequent fractions. The influence of environmental factors (humidity, sweat, changes in skin temperature) or of actual contact between the bolus and the patient’s skin was not analysed. Finally, the comparison included only two materials – ABS and TPC – without reference to TPU or PLA, which are more widely described in the literature.

In the next part of the project (Part II), we plan to analyse bolus adhesion to the skin and assess the impact of observed material changes on the actual dose distribution. It will also be essential to verify stability after repeated irradiation and simulate clinical conditions (skin contact, moisture, temperature fluctuations). In the long run, dosimetric assessment will be crucial in therapy, where even minor deviations in bolus adjustment can affect target volume coverage and the protection of critical structures. In Part II, these aspects will be addressed through dose-distribution measurements (using phantoms), quantitative adhesion and bolus–skin contact tests, repeated-irradiation experiments simulating multi-fraction treatment, and controlled phantom studies assessing positioning stability and air-gap formation.

## Conclusion

ABS showed measurable radiation-induced effects, including a 0.05 mm dimensional change, a ~ 3 °C decrease in Tg, and a 70% reduction in friction. At the same time, TPC exhibited only minimal variations across mechanical, chemical, and surface properties. These results clearly indicate that TPC exhibits greater chemical, thermal, and mechanical resistance to therapeutic radiation than ABS. In practice, this may translate into better adaptation to the patient’s surface, reduced susceptibility to degradation, and more reproducible irradiation conditions in subsequent radiotherapy fractions. Both materials can be used clinically, but TPC appears to be a more promising candidate for further research and potential clinical implementation. Further clinical validation—particularly assessing bolus–skin adhesion, positional stability, and dosimetric impact during multi-fraction radiotherapy—is necessary before widespread implementation. In the short term, the results may support more informed material selection for 3D-printed boluses and help clinicians anticipate the behaviour of ABS and TPC during treatment. In the longer term, improved understanding of radiation-induced material changes may contribute to the development of more durable, reliable and patient-specific bolus solutions in radiotherapy.

## Supplementary Information

Supplementary Information.

## Data Availability

The data supporting the reported results are available at 10.7910/DVN/VG2QTB (accessed September 3, 2025).

## Abbreviations

ABS: Acrylonitrile–butadiene–styrene
TPC: Thermoplastic copolyester elastomer
TPU: Thermoplastic polyurethane
DSC: Differential scanning calorimetry
FTIR: Fourier-transform infrared spectroscopy
ATR: Attenuated total reflectance
CoF: Coefficient of friction
Sa: Arithmetic mean height
Sq: Root mean square height
Sz: Maximum height
Ssk: Skewness
Sku: Kurtosis
