# Supplementary material for: Structural and physicochemical stability of 3D-printed bolus materials used in radiotherapy
Source: Sci Rep. 2026 Jan 29;16:6611. doi: 10.1038/s41598-026-36952-x (PMC12913660; doi:10.1038/s41598-026-36952-x)
Supplement: Supplementary file 1 — Supplementary Information. [file 41598_2026_36952_MOESM1_ESM.docx]

Radiation-induced physicochemical changes in 3D-printed bolus materials for radiotherapy: Part 1 – surface degradation and structural stability

**Karolina Jezierska^1^, Martin Borůvka^2^, Martina Ryvolová^3^, Adam Hotař^3^, Helena Gronwald^4^, Magdalena Łukowiak^1^, Piotr Rawojć^1^, Helena Rudnicka^5^, Totka Bakalova^3^**

^1^ Department of Medical Physics, Pomeranian Medical University in Szczecin, ul. Ku Słońcu 13, 71-073 Szczecin, Poland

^2^ Department of Engineering Technology, Faculty of Mechanical Engineering, Technical University of Liberec, Studentska 2, 461 17 Liberec 1, Czech Republic

^3^ Department of Material Science, Faculty of Mechanical Engineering, Technical University of Liberec, Studentska 2, 461 17 Liberec, Czech Republic

^4^ Department of Propedeutics, Physical Diagnostics and Dental Physiotherapy, Pomeranian Medical University in Szczecin, al. Powstańców Wielkopolskich 72, 70-111 Szczecin, Poland

^5^ Department of Genetics and Pathology, Pomeranian Medical University in Szczecin, ul. Unii Lubelskiej 1, 71-252 Szczecin, Poland

***** Correspondence: karolina.jezierska@pum.edu.pl


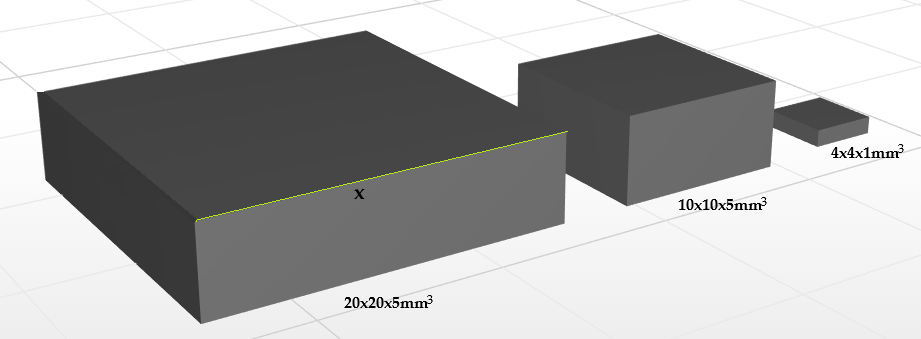
**Figure S1.** Sample models prepared for testing, showing three variants of 3D print dimensions. The x and y designations refer to the measured lengths of the sample sides.


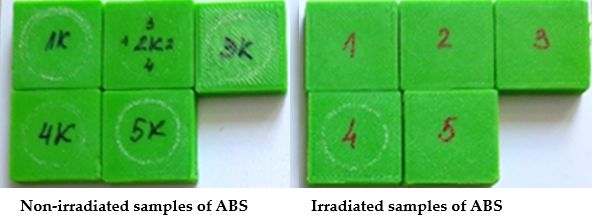


**Figure S2.** Tested samples of ABS after tribological evaluation. Samples with and without letter K are non-irradiated and irradiated samples, respectively.


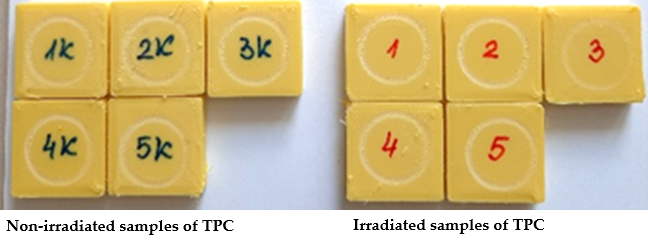


**Figure S3.** Tested samples of TPC after tribological evaluation. Samples with and without letter K are non-irradiated and irradiated samples, respectively.


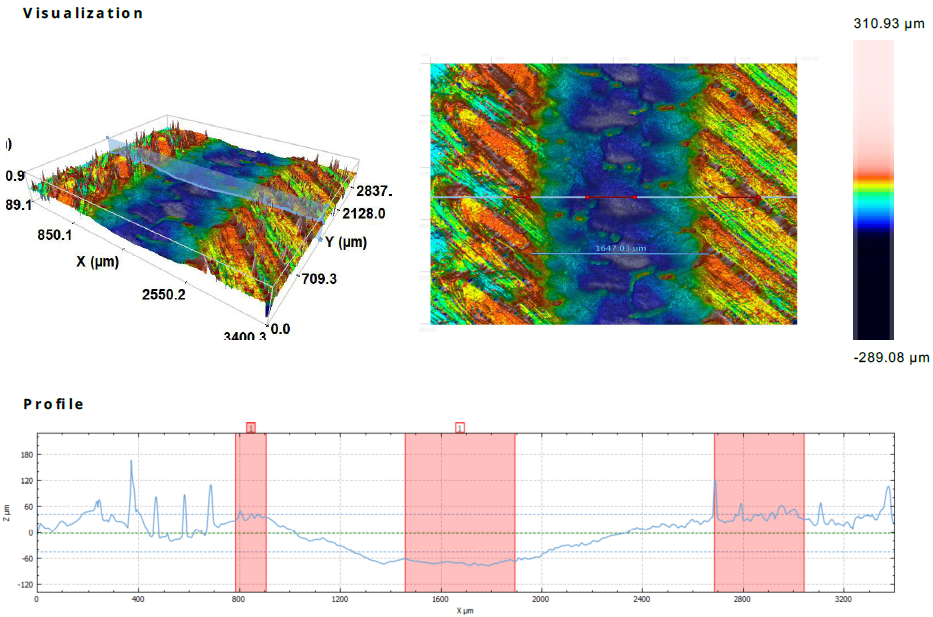


**Figure S4.** Example of confocal microscopy analysis of wear track after tribological testing, showing 3D surface topography (left), 2D surface map with track width measurement (right), and wear profile depth (bottom). The colour scale on the right indicates surface height in micrometres.

**
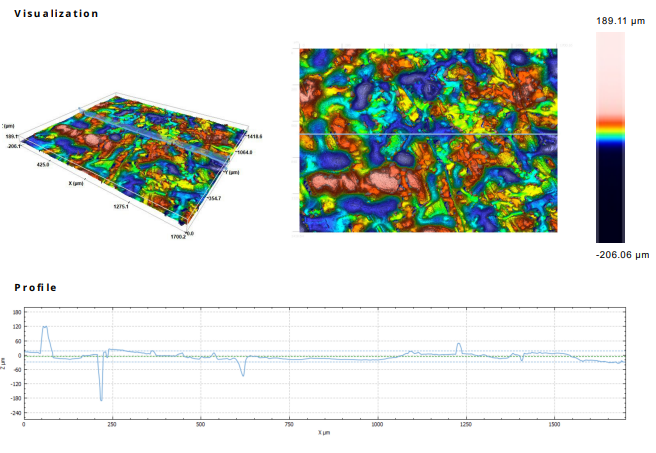
**

**Figure S5.** Example of surface topography analysis performed using confocal microscopy: three-dimensional surface map (left), two-dimensional height distribution map (middle), and height profile along the selected measurement line (bottom). The colour scale on the right represents surface height in micrometres.

**
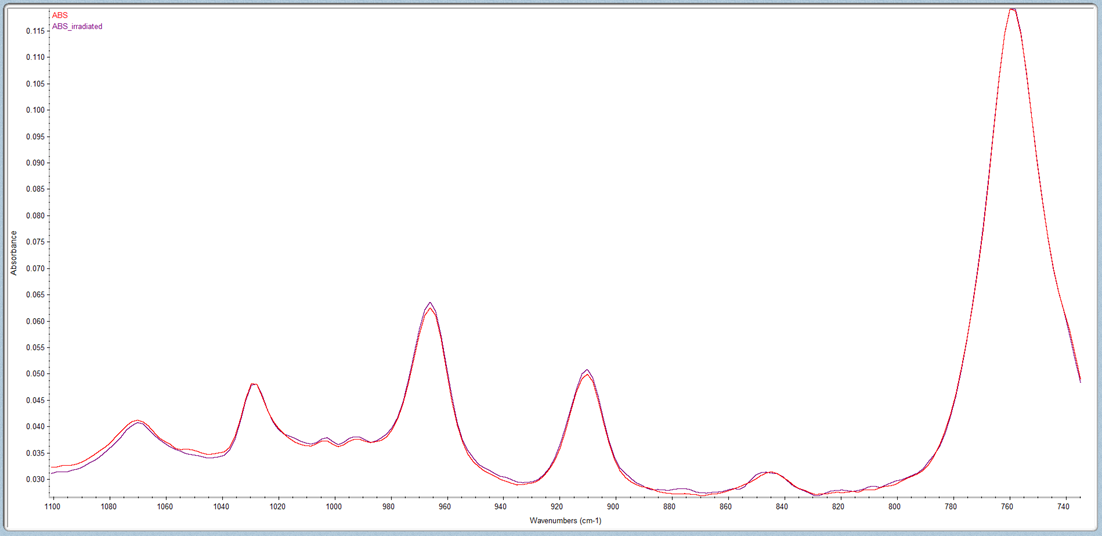
Figure S6.** FTIR spectra of ABS before (red) and after (purple) irradiation, in the range of 1100–740 cm⁻¹, covering characteristic bands for butadiene units.


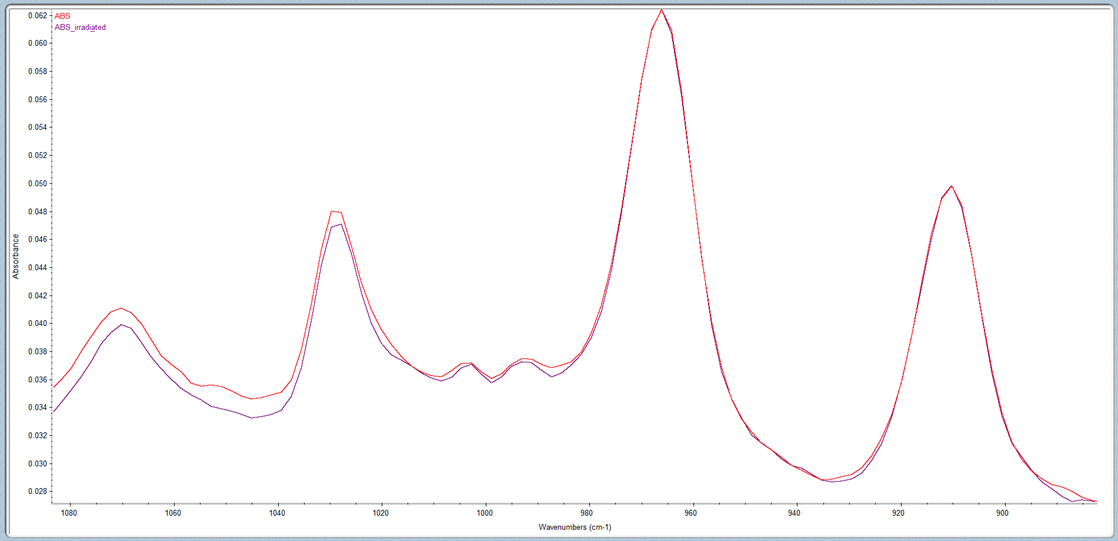


**Figure S7.** FTIR spectra of ABS before (red) and after (purple) irradiation, in the range of 1100–840 cm⁻¹, covering characteristic bands for butadiene units.


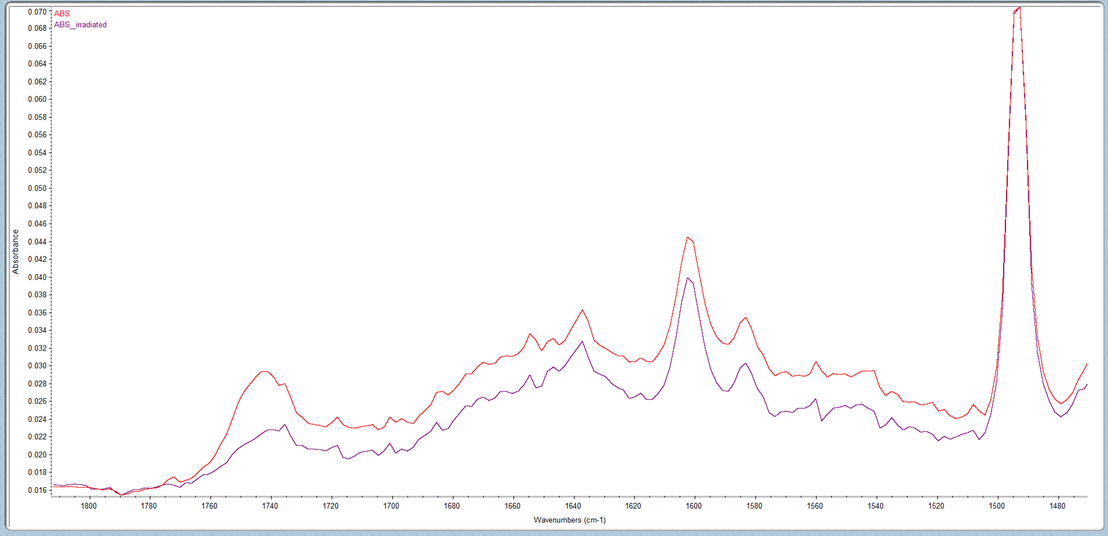
**Figure S8.** FTIR spectra of ABS before (red) and after (purple) irradiation, in the range of 1800–1480 cm⁻¹, including the carbonyl band and the vinyl band, which is often used for mapping butadiene units due to reduced overlap with other peaks.


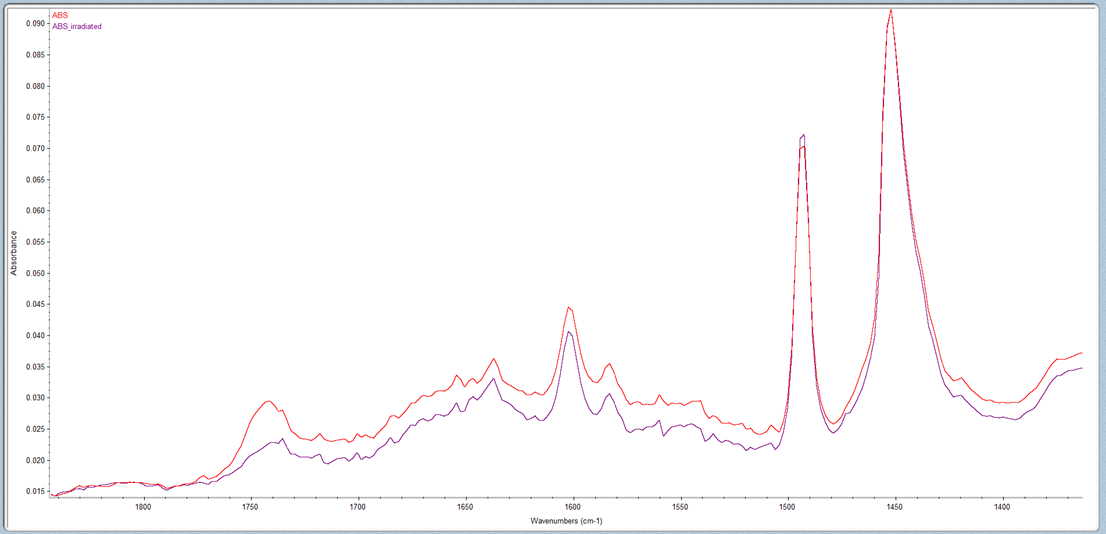
**Figure S9.** FTIR spectra of ABS before (red) and after (purple) irradiation, in the range of 1800–1400 cm⁻¹. A decrease in the intensity of carbonyl bands was observed, along with noticeable differences in the 1600–1500 cm⁻¹ region associated with aromatic and C=C stretching vibrations.


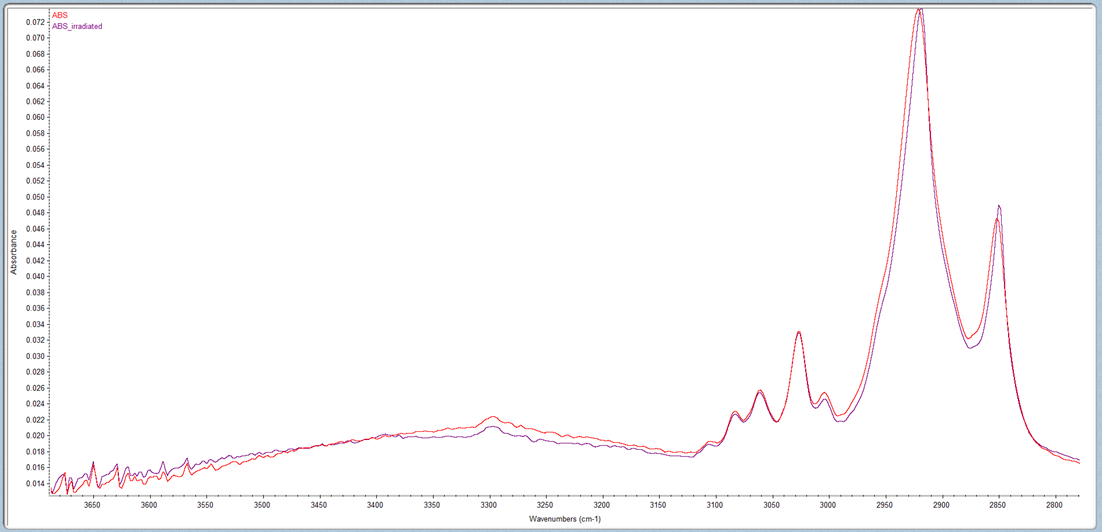


**Figure S10.** FTIR spectra of ABS before (red) and after (purple) irradiation, in the range of 3700–2800 cm⁻¹. Characteristic C–H stretching vibrations are visible, along with a broad band around 3400 cm⁻¹ attributed to hydroxyl groups, which showed decreased intensity after irradiation.


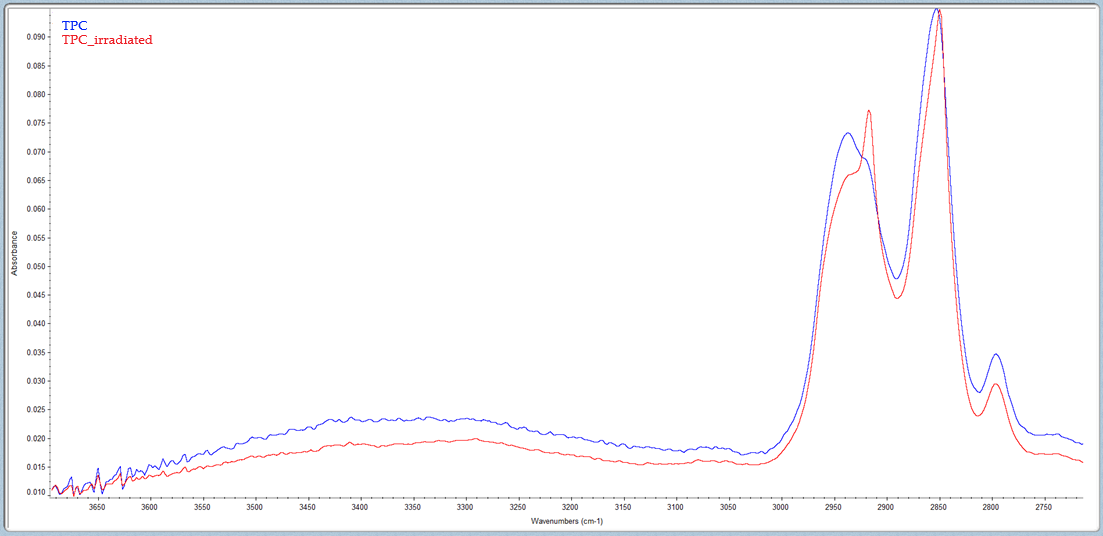
**Figure S11.** FTIR spectra of TPC before (blue) and after (red) irradiation, in the range of 3700–2700 cm⁻¹. Characteristic C–H stretching bands are visible. After irradiation, a decrease in the broad band around 3400 cm⁻¹ (associated with hydroxyl groups) was observed, along with slight variations in C–H band intensities.


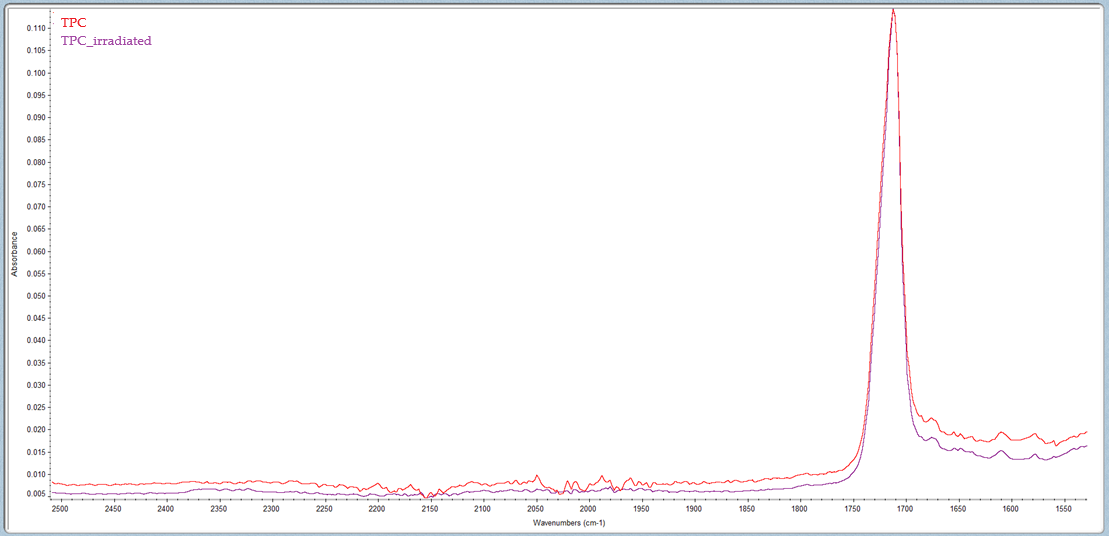
**Figure S12.** FTIR spectra of TPC before (red) and after (purple) irradiation in the range of 2500–1550 cm⁻¹. The most characteristic feature is the strong absorption around ~1715 cm⁻¹, attributed to C=O stretching vibrations. After irradiation, a decrease in intensity and broadening of this band were observed.


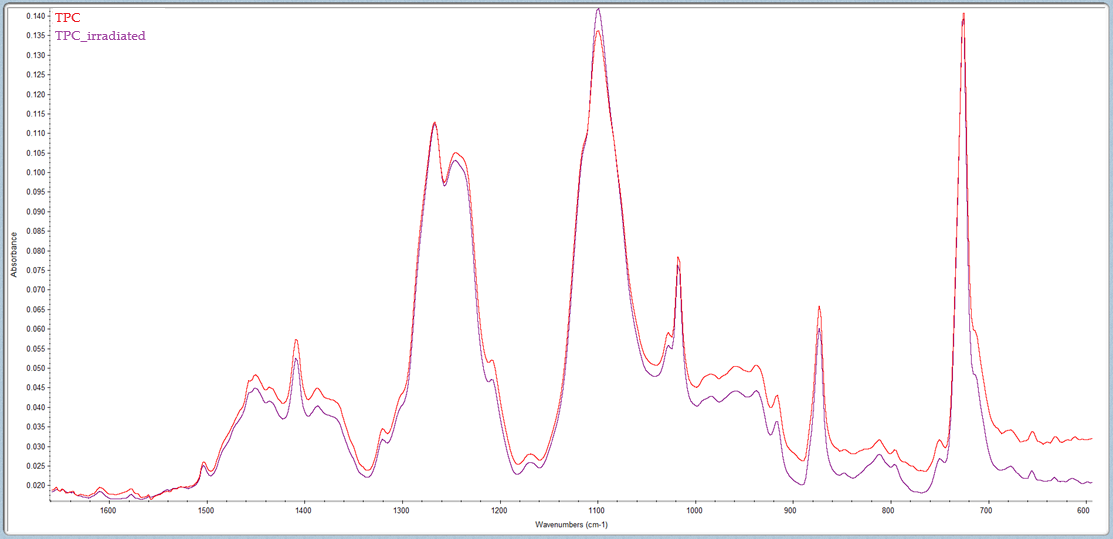


**Figure S13.** FTIR spectra of TPC before (red) and after (purple) irradiation, in the range of 1600–600 cm⁻¹. Characteristic bands corresponding to C–H and C–C stretching vibrations are visible. After irradiation, decreased intensity was observed around ~1450–1370 cm⁻¹ and changes in the 1100–700 cm⁻¹ region.

**Table S1.** Selected mechanical and thermal properties of ABS and TPC filaments before exposure to X-ray radiation. Data based on manufacturers' technical data sheets.

| Material parameter | ABS | TPC |
| --- | --- | --- |
| Filament diameter [mm] | 1.75 | 1.75 |
| Density [g/cm³] | 1.18 | 1.15 |
| Shore hardness D | 73.4 | 40 |
| Softening Point [°C] | 98/100 | 80 |
| The glass transition temperature [°C] | 106.4 | No data |

**Table S2.** FDM 3D printing parameters (Zortrax M300 DUAL) for ABS and TPC filaments according to the manufacturer's information and preliminary optimisation performed as part of this study.

| Printing parameter | ABS | TPC |
| --- | --- | --- |
| Filament diameter [mm] | 1.75 | 1.75 |
| The printing (nozzle) temperature [°C] | 260 | 230 |
| Print speed [mm/s] | 100% | 50% |
| Layer thickness [mm] | 0.15 | 0.15 |
| Infill density [%] | 90 | 90 |
| Supports | No | No data |

**Table S3.** Statistical data for dimension x [mm], for ABS and TPC polymer samples.

|  | | x [mm] | | | | | | |
| --- | --- | --- | --- | --- | --- | --- | --- | --- |
| Material | **Radiation** | **Mean** | **Median** | **Minimum** | **Maximum** | **25th Percentile** | **75th Percentile** | **Standard deviation** |
| ABS | before | 20.58 | 20.56 | 20.46 | 20.71 | 20.53 | 20.66 | 0.07 |
|  | after | 20.53 | 20.53 | 20.42 | 20.70 | 20.48 | 20.59 | 0.07 |
| TPC | before | 20.25 | 20.26 | 20.09 | 20.41 | 20.22 | 20.28 | 0.08 |
|  | after | 20.27 | 20.28 | 20.10 | 20.40 | 20.24 | 20.31 | 0.08 |

**Table S4.** Results of the Shapiro–Wilk normality test for dimension x [mm] for ABS and TPC polymer samples, before and after irradiation. W = Shapiro–Wilk statistic, p = p-value

| Material | n | x [mm] | | | |
| --- | --- | --- | --- | --- | --- |
|  |  | **Before irradiation** | | **After irradiation** | |
|  |  | **W** | ***p*** | **W** | ***p*** |
| ABS | 25 | 0.92 | 0.07 | 0.94 | 0.13 |
| TPC | 25 | 0.94 | 0.16 | 0.90 | 0.02 |

**Table S5.** *p*-values and test statistic from statistical comparisons of dimension x[mm] of samples of ABS and TPC polymer before and after irradiation. p = p-value, t = t-value (Student’s t-test), and U = U-value (Mann–Whitney U test).

| Material | x [mm] | |
| --- | --- | --- |
|  |  |  |
|  | **t/U** | ***p*** |
| ABS | t(48)=2.37 | 0.02 |
| TPC | U=237 | 0.15 |

**Table S6**. Statistical data for the average value of the coefficient of friction for ABS and TPC polymer samples.

|  | | The average value of the coefficient of friction | | | | | | |
| --- | --- | --- | --- | --- | --- | --- | --- | --- |
| Material | **Radiation** | **Mean** | **Median** | **Minimum** | **Maximum** | **25th Percentile** | **75th Percentile** | **Standard deviation** |
| ABS | Non-irradiated | 0.396 | 0.396 | 0.359 | 0.439 | 0.380 | 0.405 | 0.030 |
|  | irradiated | 0.133 | 0.091 | 0.075 | 0.294 | 0.082 | 0.125 | 0.092 |
| TPC | Non-irradiated | 0.837 | 0.835 | 0.815 | 0.854 | 0.829 | 0.852 | 0.016 |
|  | irradiated | 0.735 | 0.764 | 0.525 | 0.817 | 0.760 | 0.809 | 0.120 |

**Table S7.** Results of the Shapiro-Wilk normality test for the average value of the coefficient of friction for ABS and TPC polymer non-irradiated and radiated samples. p = p-value, W = Shapiro–Wilk statistic.

| Material | n | The average value of the coefficient of friction | | | |
| --- | --- | --- | --- | --- | --- |
|  |  | **Non-irradiated** | | **Irradiated** | |
|  |  | **W** | ***p*** | **W** | ***p*** |
| ABS | 5 | 0.99 | 0.96 | 0.72 | 0.02 |
| TPC | 5 | 0.93 | 0.59 | 0.73 | 0.02 |

**Table S8.** *p*-values and test statistic from statistical comparisons of the average value of the coefficient of friction of non-irradiated and irradiated samples of ABS and TPC. p = p-value and U = U-value (Mann–Whitney U test).

| Material | The average value of the coefficient of friction | |
| --- | --- | --- |
|  |  |  |
|  | **U** | ***p*** |
| ABS | 0 | 0.01 |
| TPC | 1 | 0.02 |

|  | | | Width and depth of the wear rate for TPC | | | | | | | |
| --- | --- | --- | --- | --- | --- | --- | --- | --- | --- | --- |
| parameter | **Radiation** | **Mean** | | **Median** | **Minimum** | **Maximum** | **25th Percentile** | **75th Percentile** | **Standard deviation** |  |
| Width [mm] | Non-irradiated | 1.562 | | 1.567 | 1.466 | 1.647 | 1.510 | 1.613 | 0.052 |  |
|  | irradiated | 1.484 | | 1.470 | 1.457 | 1.540 | 1.459 | 1.510 | 0.028 |  |
| Depth [µm] | Non-irradiated | 106.992 | | 105.670 | 80.090 | 136.540 | 91.575 | 122.410 | 15.418 |  |
|  | irradiated | 116.083 | | 107.875 | 92.960 | 155.620 | 95.625 | 136.540 | 20.458 |  |

**Table S9.** Statistical data for the width and depth of the wear rate for TPC polymer samples.

**Table S10.** *p*-values and test statistic from statistical comparisons of the width and depth of the wear rate for TPC polymer non-irradiated and radiated samples. p = p-value and U = U-value (Mann–Whitney U test).

| Material | Width and depth of the wear rate for TPC | |
| --- | --- | --- |
|  |  |  |
|  | **U** | ***p*** |
| Width [mm] | 2 | 0.11 |
| Depth [µm] | 7 | 0.89 |

**Table S11.** Results of the Shapiro–Wilk normality test for the surface roughness parameters for ABS polymer samples, before and after irradiation. p = p-value and W = Shapiro–Wilk statistic.

| parameters | n | Surface roughness parameters for ABS | | | |
| --- | --- | --- | --- | --- | --- |
|  |  | **Before irradiation** | | **After irradiation** | |
|  |  | **W** | ***p*** | **W** | ***p*** |
| Sa | 15 | 0.98 | 0.98 | 0.96 | 0.73 |
| Sku | 15 | 0.92 | 0.21 | 0.88 | 0.05 |
| Sp | 15 | 0.83 | 0.01 | 0.92 | 0.19 |
| Sq | 15 | 0.98 | 0.96 | 0.92 | 0.18 |
| Sz | 15 | 0.95 | 0.51 | 0.94 | 0.44 |
| Sv | 15 | 0.96 | 0.64 | 0.96 | 0.68 |
| Ssk | 15 | 0.89 | 0.06 | 0.85 | 0.02 |

**Table S12.** Results of the Shapiro–Wilk normality test for the surface roughness parameters for TPC polymer samples, before and after irradiation. p = p-value and W = Shapiro–Wilk statistic.

| parameters | n | Surface roughness parameters for TPC | | | |
| --- | --- | --- | --- | --- | --- |
|  |  | **Before irradiation** | | **After irradiation** | |
|  |  | **W** | ***p*** | **W** | ***p*** |
| Sa | 15 | 0.94 | 0.34 | 0.88 | 0.05 |
| Sku | 15 | 0.96 | 0.71 | 0.96 | 0.77 |
| Sp | 15 | 0.98 | 0.96 | 0.94 | 0.33 |
| Sq | 15 | 0.94 | 0.36 | 0.89 | 0.07 |
| Ssk | 15 | 0.96 | 0.68 | 0.97 | 0.87 |
| Sv | 15 | 0.96 | 0.62 | 0.91 | 0.14 |
| Sz | 15 | 0.95 | 0.60 | 0.96 | 0.62 |

**Table S13.** *p*-values and test statistic from statistical comparisons of surface roughness parameters for samples of ABS and TPC polymer before and after irradiation. p = p-value, t = t-value (Student’s t-test), and U = U-value (Mann–Whitney U test).

| Parameter | Surface roughness parameters for samples of | | | | | |
| --- | --- | --- | --- | --- | --- | --- |
|  | ABS | | | **TPC** | | |
|  | **t/U** | ***p*** | **t** | | ***p*** |  |
| Sa | t(28)=1.68 | 0.10 | t(28)=2.35 | | 0.03 |  |
| Sku | U=17 | <0.01 | t(28)=1.50 | | 0.14 |  |
| Sp | U=10 | <0.01 | t(28)=3.98 | | <0.01 |  |
| Sq | t(28)=2.67 | 0.01 | t(28)=2.47 | | 0.02 |  |
| Sz | t(28)=5.60 | <0.01 | t(28)=3.96 | | <0.01 |  |
| Sv | t(28)=4.02 | <0.01 | t(28)=2.41 | | 0.02 |  |
| Ssk | U=82 | 0.21 | t(28)=0.91 | | 0.37 |  |

**Table S14.** Statistical data for the Shore D hardness for ABS and TPC polymer samples.

|  | | Shore D hardness | | | | | | |
| --- | --- | --- | --- | --- | --- | --- | --- | --- |
| Material | **Radiation** | **Mean** | **Median** | **Minimum** | **Maximum** | **25th Percentile** | **75th Percentile** | **Standard deviation** |
| ABS | before | 78 | 77 | 74 | 88 | 75 | 80 | 3 |
|  | after | 80 | 80 | 76 | 83 | 79 | 81 | 2 |
| TPC | before | 38 | 38 | 35 | 41 | 37 | 40 | 2 |
|  | after | 41 | 41 | 38 | 43 | 40 | 42 | 1 |

**Table S15.** Results of the Shapiro–Wilk normality test for the average value of Shore D hardness for ABS and TPC polymer samples, before and after irradiation. p = p-value and W = Shapiro–Wilk statistic.

| Material | n | Shore D hardness | | | |
| --- | --- | --- | --- | --- | --- |
|  |  | **Before irradiation** | | **After irradiation** | |
|  |  | **W** | ***p*** | **W** | ***p*** |
| ABS | 25 | 0.87 | <0.01 | 0.95 | 0.22 |
| TPC | 25 | 0.91 | 0.04 | 0.94 | 0.13 |

**Table S16.** *p*-values and test statistic from statistical comparisons of Shore D hardness of samples of ABS and TPC polymer before and after irradiation. p = p-value and U = U-value (Mann–Whitney U test).

| Material | Shore D hardness | |
| --- | --- | --- |
|  |  |  |
|  | **U** | ***p*** |
| ABS | 167 | <0.01 |
| TPC | 71 | <0.01 |
